# Integrating Drug Repurposing into EU Health Crisis Preparedness: The Strategic Role of Health Emergency Preparedness and Response Authority (HERA)

**DOI:** 10.3390/pharmacy14030072

**Published:** 2026-05-12

**Authors:** Atanas Toshev, Stanislav Gueorguiev, Anna Mihaylova, Violeta Getova-Kolarova, Vasil Madzharov, Dimitar Mirchev, Elina Petkova-Gueorguieva

**Affiliations:** 1 Department of Organisation and Economics of Pharmacy, Faculty of Pharmacy, Medical University of Plovdiv, 4002 Plovdiv, Bulgaria; stanislav.georgiev@mu-plovdiv.bg (S.G.);; 2 Department of Health Care Management, Faculty of Public Health, Medical University of Plovdiv, 4002 Plovdiv, Bulgaria; anna.mihaylova@mu-plovdiv.bg; 3 Department of Organisation and Economics of Pharmacy, Faculty of Pharmacy, Medical University of Sofia, 1431 Sofia, Bulgaria; v.getova@pharmfac.mu-sofia.bg; 4 Department of Language and Specialized Training, Medical University of Plovdiv, 4002 Plovdiv, Bulgaria; dimitar.mirchev@mu-plovdiv.bg; 5 Department of Health Policy and Management, Faculty of Public Health, Medical University of Sofia, 1431 Sofia, Bulgaria; e.petkova@foz.mu-sofia.bg

**Keywords:** HERA, drug repurposing, EU Health Union, medical countermeasures, pandemic preparedness, pharmaceutical resilience

## Abstract

The COVID-19 pandemic exposed significant vulnerabilities in the European Union’s health security architecture and highlighted the need for stronger coordination mechanisms for managing cross-border health threats. In response, the European Union established the Health Emergency Preparedness and Response Authority (HERA) as a central body responsible for strengthening preparedness, coordinating procurement, and supporting the development and availability of medical countermeasures. This study examines the potential role of drug repurposing as a strategic tool within the evolving EU health crisis preparedness framework. A narrative literature review and policy analysis were conducted using scientific publications indexed in PubMed and Scopus, as well as institutional and regulatory documents from the European Commission, the European Medicines Agency (EMA), and other relevant organisations. The findings indicate that drug repurposing offers important advantages during health emergencies, including shorter development timelines, lower research costs, and the possibility of leveraging existing manufacturing and regulatory infrastructures. At the same time, several challenges remain, particularly regarding regulatory coordination, intellectual property considerations, and the scalability of pharmaceutical production during periods of increased demand. The analysis suggests that drug repurposing could evolve from an ad hoc response mechanism into a more institutionalised component of EU health crisis preparedness. Integrating repurposing strategies into HERA’s threat prioritisation, regulatory coordination, and industrial preparedness mechanisms may significantly enhance the European Union’s ability to respond rapidly and effectively to future health emergencies.

## 1. Introduction

The COVID-19 pandemic exposed significant structural weaknesses in the European Union’s health security architecture and revealed the limitations of fragmented national responses to large-scale public health emergencies. During the early stages of the pandemic, Member States adopted largely uncoordinated strategies to secure vaccines, therapeutics, and other medical supplies, which resulted in competition for scarce resources and disruptions across pharmaceutical supply chains. These developments highlighted the need for stronger coordination mechanisms at the European level capable of anticipating and responding to cross-border health threats more effectively [1,2].

In response to these challenges, the European Union initiated a series of institutional reforms aimed at strengthening its collective preparedness and crisis response capacity. A central element of these reforms was the establishment of the Health Emergency Preparedness and Response Authority (HERA), created within the European Commission to enhance the Union’s ability to anticipate, prepare for, and respond to serious cross-border health threats. HERA was designed to function as a strategic coordination body responsible for threat intelligence, research and innovation support, procurement coordination, and the strengthening of industrial capacities for the production of medical countermeasures [1,3].

Health security has consequently emerged as a strategic priority within the broader framework of the European Health Union. The development of this initiative reflects a shift from predominantly national crisis management approaches toward a more integrated European model capable of addressing health emergencies collectively. This transformation includes the strengthening of the mandates of key EU agencies, such as the European Medicines Agency (EMA) and the European Centre for Disease Prevention and Control (ECDC), as well as the creation of new mechanisms for coordinated procurement, stockpiling of medical countermeasures, and industrial preparedness. As a result, health security is increasingly recognised not only as a public health concern but also as a critical dimension of economic resilience, strategic autonomy, and the stability of the internal market [4].

Despite these institutional developments, significant challenges remain within the European pharmaceutical landscape, particularly regarding the timely availability of effective therapeutic options during health emergencies. The development of new medicinal products typically requires lengthy research and development processes, extensive clinical trials, and complex regulatory evaluation procedures, which can take more than a decade before a novel therapy reaches the market. During rapidly evolving health crises, such timelines are often incompatible with the urgent need for therapeutic interventions. At the same time, European health systems remain vulnerable to disruptions in pharmaceutical supply chains, partly due to their reliance on global production networks for active pharmaceutical ingredients and essential medicines [5,6].

In this context, drug repurposing has attracted increasing attention as a complementary strategy for accelerating access to therapeutic options during health emergencies. Drug repurposing involves identifying new therapeutic indications for existing medicinal products that have already been approved or extensively studied. Because such medicines already possess established safety profiles, pharmacological data, and manufacturing pathways, repurposing may significantly reduce the time, cost, and risk associated with the development of new treatments. Consequently, this approach may provide a valuable mechanism for rapidly deploying therapeutic interventions in response to emerging infectious diseases or other public health emergencies [7].

Recent health crises have demonstrated both the opportunities and limitations associated with repurposing strategies. During the COVID-19 pandemic, several existing medicinal products were rapidly evaluated for potential therapeutic benefits, illustrating how repurposing can provide early treatment options while more targeted therapies and vaccines are being developed. At the same time, these experiences highlighted the importance of coordinated clinical research, regulatory oversight, and evidence-based decision-making in order to avoid ineffective or potentially harmful treatments.

Against this background, the evolving institutional architecture of EU health security raises important questions regarding the role that drug repurposing could play in future crisis preparedness strategies. As the central EU body responsible for coordinating preparedness and response to health emergencies, HERA may serve as a key institutional platform for integrating repurposing strategies into broader frameworks of medical countermeasure development, regulatory evaluation, and industrial preparedness.

### Aim of the Study

The aim of this study is to analyse the strategic role of HERA in the management of health crises within the European Union, with particular emphasis on the potential integration of drug repurposing as a policy instrument for improving the availability of medical countermeasures. By examining the institutional mechanisms developed within the EU health security framework, the study seeks to explore how repurposing strategies could contribute to strengthening preparedness, accelerating access to therapies, and enhancing the resilience of European pharmaceutical supply chains.

## 2. Materials and Methods

This study applies a narrative scoping review combined with policy analysis to examine the role of drug repurposing within the evolving framework of European Union health crisis preparedness and response. The analysis focuses on the institutional architecture surrounding the Health Emergency Preparedness and Response Authority (HERA) and its potential role in integrating drug repurposing into strategies for ensuring the availability of medical countermeasures during health emergencies.

The selection of studies involved a process of several stages that entailed the examination of titles and abstracts obtained from both PubMed and Scopus databases. Any article that failed to address any of the three topics—namely drug repurposing, EU health policy, and medical countermeasures—was discarded during the title/abstract screening phase. Further screening was carried out through full-text evaluation to establish the eligibility of the articles according to set inclusion criteria.

**Figure 1:**
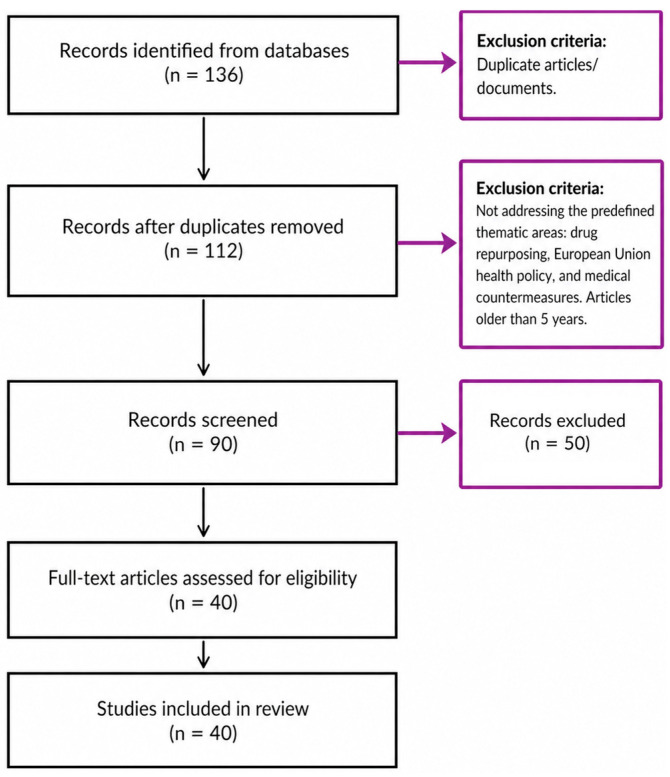
PRISMA flow diagram.

The figure illustrates the study selection process conducted in accordance with the principles of PRISMA methodology. During the identification phase, a total of 136 records were retrieved from the PubMed and Scopus databases. Prior to screening, 24 duplicate records were removed, resulting in 90 records eligible for further assessment (Figure 1).

In the screening phase, titles and abstracts were evaluated, leading to the exclusion of 50 records that did not address the predefined thematic areas, namely drug repurposing, European Union health policy, and medical countermeasures. The remaining 40 records were subsequently assessed in full text.

During the eligibility phase, all 40 articles were examined against the predefined inclusion and exclusion criteria. The inclusion criteria comprised studies and documents focusing on drug repurposing approaches, pharmaceutical policy, health emergency preparedness, and EU-level institutional arrangements. Studies and documents were excluded if they concerned non-medicinal products or lacked a policy dimension As a result of this process, 40 studies were included in the article.

The inclusion criteria involved studies concerning drug repurposing approaches, pharmaceutical policy, health emergency preparedness, and EU-based institutional arrangements. The exclusion criteria were studies on non-medical products in human medicine, research articles with no policy content, and publications without adequate methodological clarity. Despite the attempt to obtain an extensive number of relevant literature, it is important to note that a scoping review cannot be considered exhaustive due to the nature of the methodology applied. The heterogeneity of sources and lack of quality assessment could result in selection bias.

### 2.1. Literature Search Strategy

A narrative review of the scientific literature was conducted using the databases PubMed and Scopus. The search focused on publications addressing drug repurposing, pharmaceutical supply chains, and EU health crisis governance.

Search queries included combinations of the following keywords: “drug repurposing”, “medical countermeasures”, “HERA”, “EU health security”, “pandemic preparedness”, “pharmaceutical supply chains”.

Only peer-reviewed publications written in English and published between 2020 and 2025 were considered, reflecting the period following the COVID-19 pandemic when significant reforms in EU health governance were introduced. Additional relevant references were identified through backward citation tracking.

### 2.2. Policy Document Selection

In addition to the scientific literature, the analysis incorporated institutional and regulatory documents relevant to EU health crisis governance. These included:

1. European Commission communications and policy strategies related to the European Health Union and HERA;
2. EU legislative acts addressing cross-border health threats and emergency response mechanisms;
3. reports and analyses from the European Parliament and European Court of Auditors concerning the EU response to the COVID-19 pandemic;
4. guidance documents and policy reports from international organisations such as the World Health Organization (WHO) and the Organisation for Economic Co-operation and Development (OECD) addressing pharmaceutical resilience and drug repurposing strategies.

Legislative and regulatory documents were primarily identified through EUR-Lex, while institutional reports were retrieved from official European Commission and EU agency repositories.

### 2.3. Analytical Framework

The collected literature and policy documents were analysed using a thematic policy analysis approach. The analysis was structured around three interrelated analytical dimensions that reflect the lifecycle of health crisis governance within the European Union:

Preparedness—institutional mechanisms aimed at anticipating health threats, strengthening pharmaceutical resilience, and building manufacturing and stockpiling capacities.

Crisis response—operational instruments deployed during health emergencies, including procurement mechanisms, regulatory coordination, and crisis governance structures.

Drug repurposing as a medical countermeasure strategy—the potential role of repurposing existing medicines to accelerate therapeutic responses during health crises.

The thematic analysis focused on identifying how drug repurposing intersects with these three dimensions within the institutional framework coordinated by HERA.

### 2.4. Scope and Study Limitations

Given the rapidly evolving nature of EU health crisis governance, this study focuses primarily on institutional frameworks and policy mechanisms rather than quantitative evaluation of specific therapeutic interventions. The analysis therefore aims to provide a conceptual and policy-oriented assessment of the potential role of drug repurposing within the EU preparedness architecture.

## 3. Results

### 3.1. Institutional Architecture of EU Health Crisis Governance

The COVID-19 pandemic acted as a catalyst for significant institutional reforms within the European Union aimed at strengthening preparedness and response to cross-border health threats. One of the most important developments was the establishment of the Health Emergency Preparedness and Response Authority (HERA), created within the European Commission as a central coordination body responsible for anticipating, preparing for, and responding to health emergencies [1,8].

HERA was designed to address structural vulnerabilities revealed during the pandemic, particularly the lack of coordinated mechanisms for securing medical countermeasures across the European Union. Its mandate covers the full lifecycle of health crisis management, ranging from threat intelligence and preparedness planning to operational response during declared public health emergencies [9,10,11,12,13]. In its preparedness phase, HERA focuses on identifying emerging health threats, assessing vulnerabilities within pharmaceutical supply chains, supporting research and innovation for medical countermeasures, and strengthening industrial production capacities within the European Union. These activities are closely linked to the broader institutional framework of the European Health Union and complement the roles of other EU agencies such as the European Medicines Agency (EMA) and the European Centre for Disease Prevention and Control (ECDC) [4,14].

When a public health emergency is formally declared at the EU level, HERA transitions into crisis response mode. In this phase, it coordinates procurement strategies, supports the rapid deployment of medical countermeasures, and facilitates cooperation between the European Commission, Member States, and relevant European agencies [3,15].

### 3.2. Procurement and Coordination Mechanisms for Medical Countermeasures

Ensuring timely access to medical countermeasures represents a central component of EU crisis preparedness. Several procurement mechanisms have been developed to facilitate coordinated access to essential medicines, vaccines, and other health products during emergencies [16,17].

One mechanism allows the European Commission to procure medical countermeasures directly using EU budgetary instruments, acting effectively as a central purchaser on behalf of Member States. Under this model, products may subsequently be redistributed, donated, or sold to participating countries depending on the operational needs during a health crisis [18,19,20].

A second mechanism involves joint procurement procedures, which enable multiple Member States to collectively negotiate and purchase medical countermeasures through coordinated procurement processes. Participation in these procedures is voluntary but allows countries to benefit from stronger negotiating power and improved access to critical health products during periods of high global demand [21].

A third procurement model may be activated during formally declared public health emergencies at the EU level. Under this framework, Member States may mandate the European Commission to conduct centralised procurement procedures on their behalf. This approach was inspired by the advance purchase agreements used during the COVID-19 vaccination campaign and aims to ensure rapid and coordinated acquisition of critical medical countermeasures across the Union [3].

### 3.3. Industrial Preparedness and Manufacturing Capacity

Beyond procurement mechanisms, strengthening pharmaceutical manufacturing capacity has become a key priority in the EU’s health crisis preparedness strategy. During the COVID-19 pandemic, shortages in manufacturing capacity significantly limited the availability of vaccines and other critical health products.

To address this vulnerability, the European Union established EU FAB, a network of manufacturing facilities designed to maintain readiness for rapid production scale-up in the event of future health crises. The network includes production capacities based on several vaccine technology platforms, including mRNA, viral vector, and protein-based technologies [22,23,24].

The purpose of EU FAB is to ensure that manufacturing facilities remain operationally prepared to rapidly switch to the production of vaccines or other biological medical countermeasures when a new health threat emerges. In principle, production could begin within weeks following the identification of a suitable vaccine candidate or therapeutic intervention, provided that regulatory approval is obtained.

In parallel, the European Union introduced financial instruments such as HERA Invest, designed to support research, innovation, and industrial capacity development related to medical countermeasures. These investments aim to strengthen Europe’s strategic autonomy in pharmaceutical production while reducing reliance on external supply chains during global health emergencies [25].

### 3.4. Drug Repurposing as a Medical Countermeasure Strategy

Drug repurposing has emerged as an important strategy for accelerating access to therapeutic options during health crises. By identifying new therapeutic uses for existing medicines, repurposing allows health systems to leverage previously established safety data, regulatory approvals, and manufacturing infrastructure.

During the COVID-19 pandemic, several existing medicinal products were investigated as potential therapeutic options. One of the most prominent examples was dexamethasone, a widely used corticosteroid that demonstrated significant clinical benefits in severely ill patients requiring respiratory support. Clinical trials showed that dexamethasone reduced mortality among hospitalised COVID-19 patients requiring oxygen therapy or mechanical ventilation [26].

Another example is remdesivir, originally developed as an antiviral treatment for Ebola virus infections. Although its effectiveness against COVID-19 was subject to ongoing clinical evaluation, the drug received conditional marketing authorization within the European Union, illustrating how repurposing strategies can provide early therapeutic options during rapidly evolving health emergencies [26].

Drug repurposing has also played a role in responses to other emerging health threats. During the mpox outbreak in 2022, medical countermeasures originally developed for smallpox were repurposed to support outbreak control efforts. These included vaccines and antiviral treatments that were already part of strategic medical stockpiles maintained by several countries [24].

### 3.5. Integration of Repurposing Within EU Crisis Preparedness

Within the evolving EU health crisis governance framework, drug repurposing is increasingly viewed not merely as an ad hoc scientific approach but as a strategic element of preparedness planning [27,28]. By mapping existing therapeutic classes against high-risk pathogens identified through threat prioritisation exercises, European institutions may be able to identify potential repurposing candidates before a crisis occurs [18,29].

Such proactive identification of repurposing opportunities may shorten the time required to deploy therapeutic interventions during the early stages of future health emergencies [30]. In this context, HERA plays a potential coordinating role by linking threat intelligence, regulatory expertise, industrial preparedness, and procurement strategies across the European health security architecture.

**Figure 2:**
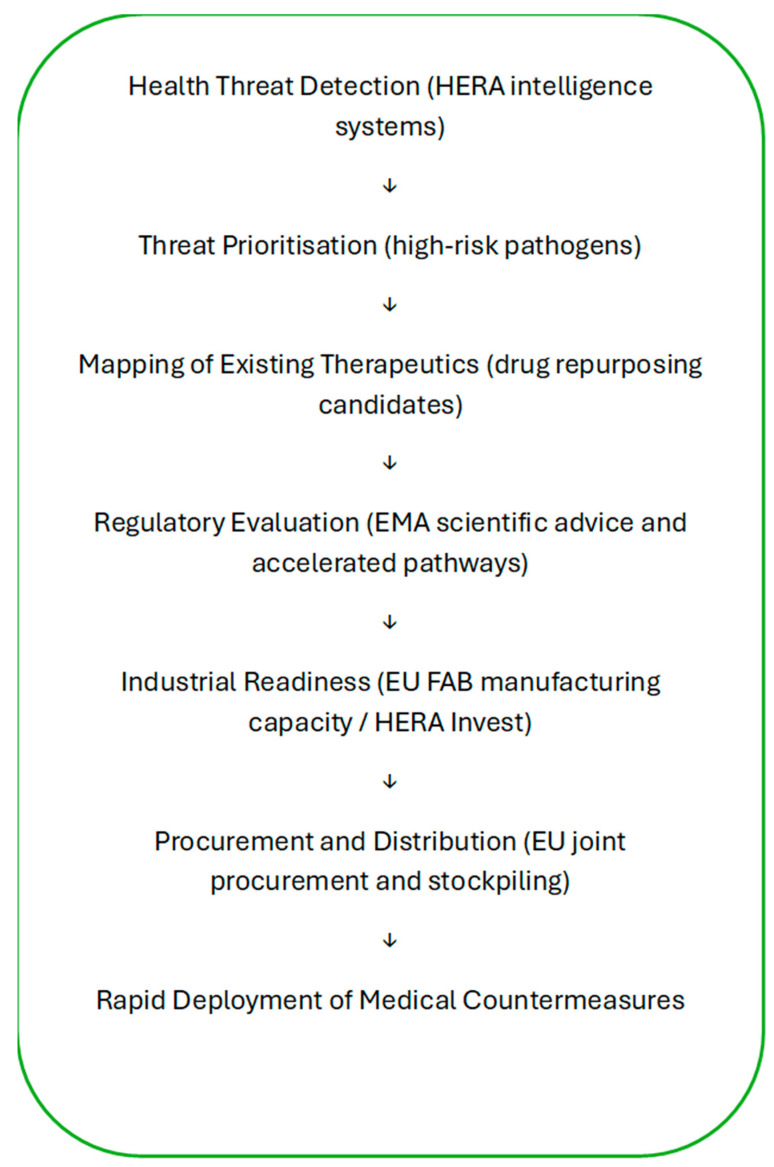
Conceptual framework for integrating drug repurposing into EU health crisis preparedness.

The integration of drug repurposing into EU crisis preparedness mechanisms can be conceptualised as a multi-stage governance process linking threat detection, regulatory evaluation, and industrial capacity (Figure 2).

The figure illustrates how drug repurposing may be integrated within the European Union’s health crisis governance architecture. The process begins with threat detection and prioritisation coordinated through HERA, followed by the identification of potential therapeutic candidates among existing medicines [27,28]. Regulatory evaluation by the European Medicines Agency (EMA), industrial preparedness mechanisms such as EU FAB, and coordinated procurement instruments enable the rapid deployment of repurposed medicines as medical countermeasures during health emergencies.

## 4. Discussion

### 4.1. Strategic Value of Drug Repurposing in Health Crisis Preparedness

The findings of this study suggest that drug repurposing may play an important role within the evolving European framework for health crisis preparedness. Compared with the traditional development of novel medicinal products, repurposing offers a faster and potentially more cost-effective pathway for identifying therapeutic options during rapidly emerging health threats. Because repurposed medicines are typically based on compounds with established safety profiles and existing manufacturing infrastructure, they may provide an early therapeutic response while new treatments and vaccines are still under development [25].

The experience of the COVID-19 pandemic illustrates both the potential and the limitations of repurposing strategies. In several cases, existing medicinal products were rapidly evaluated for potential therapeutic benefits. The identification of dexamethasone as an effective treatment for severely ill COVID-19 patients demonstrated how repurposing can deliver significant clinical impact within a relatively short timeframe. At the same time, other candidate therapies failed to demonstrate clear benefits, highlighting the need for coordinated clinical research and robust regulatory oversight in order to ensure evidence-based therapeutic decisions [26].

From a policy perspective, drug repurposing can therefore be viewed as a complementary strategy rather than a substitute for pharmaceutical innovation. Its primary value lies in shortening the time required to identify potentially effective treatments during the early phases of a health crisis, when therapeutic options are limited and uncertainty is high.

**Figure 3:**
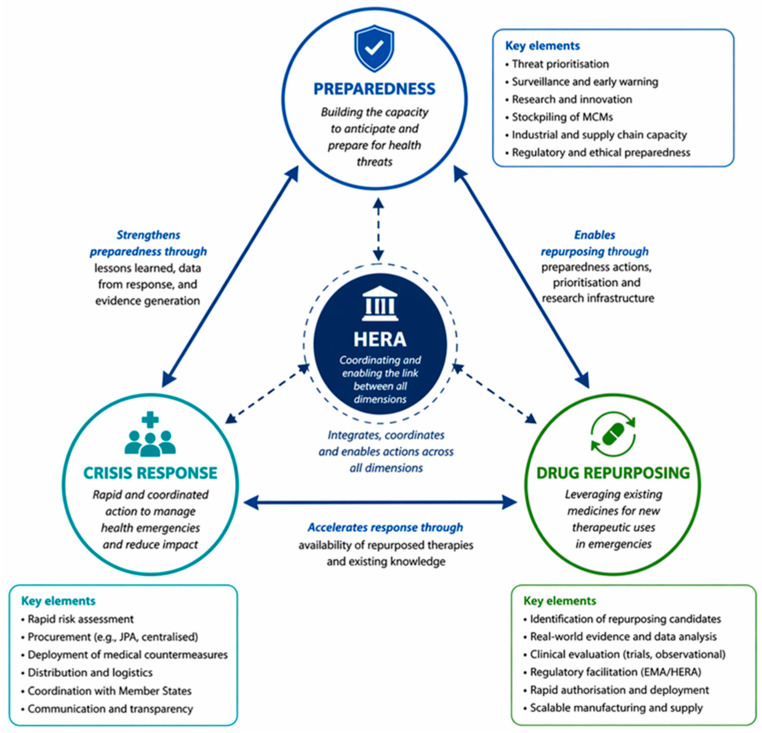
Analytical framework of EU health crisis governance and the role of drug repurposing.

Figure 3 represents the conceptual framework of health crisis management in the European Union in which drug repurposing is integrated. The structure is centred around three pillars—preparedness, crisis response, and drug repurposing. At the centre of this system is the Health Emergency Preparedness and Response Authority (HERA), which plays a leading role and connects these three elements into a single functioning architecture (Figure 3).

The coordination between the three dimensions is dynamic and bidirectional—activities related to preparedness create the conditions for more effective drug repurposing, which in turn accelerates the response to an ongoing crisis, generating additional data that further improves the preparedness phase. This framework illustrates how an integrated approach could lead to a more robust, resilient, and effective system for health security in the European Union.

### 4.2. Regulatory and Intellectual Property Challenges

Despite its potential advantages, the systematic use of drug repurposing within EU health crisis preparedness strategies faces several regulatory and legal challenges. One of the most significant barriers concerns the intellectual property framework governing medicinal products. In many cases, repurposing involves medicines that are no longer protected by patents, which can facilitate rapid access and affordability. However, repurposing may also involve products that remain subject to regulatory exclusivity or patent protection for specific therapeutic indications [31].

This creates potential tensions between public health objectives and the incentives required to stimulate pharmaceutical innovation. During the COVID-19 pandemic, debates emerged regarding access to antiviral medicines and the extent to which intellectual property rights might limit the rapid deployment of therapeutic options. Addressing these tensions will require careful policy design that balances innovation incentives with the need for equitable access to essential medicines during public health emergencies.

Another important regulatory challenge concerns the generation of robust clinical evidence. Although repurposed medicines benefit from existing safety data, their effectiveness for new indications must still be demonstrated through appropriate clinical studies. Coordinated regulatory guidance and adaptive clinical trial designs may therefore be necessary to accelerate the evaluation of repurposed therapies during future crises [32].

### 4.3. Industrial and Supply Chain Constraints

Industrial and supply chain factors also represent important constraints on the large-scale implementation of repurposing strategies. Even when a medicine is already authorised and clinically well understood, the rapid increase in global demand during a health emergency may exceed existing manufacturing capacities [33,34].

The COVID-19 pandemic demonstrated how sudden surges in demand for specific medicines can lead to supply shortages and disruptions in pharmaceutical supply chains. In some cases, medicines repurposed for pandemic treatment created competition for existing patient populations that relied on the same therapies for other medical conditions. Such situations highlight the importance of integrating repurposing strategies with broader pharmaceutical supply chain monitoring and shortage prevention mechanisms [35,36,37].

Within this context, industrial preparedness initiatives coordinated at the EU level may play a crucial role [38,39]. Mechanisms such as EU FAB and financial instruments like HERA Invest aim to strengthen manufacturing flexibility and ensure that production capacities can be rapidly adapted during health crises. Although these initiatives have primarily focused on vaccines and biological products, their underlying logic may also support the scalable production of repurposed medicines when appropriate manufacturing pathways are available [22,40].

### 4.4. Integrating Drug Repurposing into EU Health Crisis Governance

The results of this analysis indicate that drug repurposing could become a more structured component of the European Union’s health crisis preparedness strategy. Rather than emerging only as an ad hoc scientific response during emergencies, repurposing could be integrated into preparedness planning through systematic identification of potential therapeutic candidates.

Within the institutional architecture of the European Health Union, HERA appears particularly well positioned to facilitate this integration. By linking threat intelligence, regulatory coordination, pharmaceutical research, and industrial preparedness, HERA may serve as a central platform for coordinating repurposing initiatives across the European Union.

One possible avenue for strengthening this integration would be the development of a coordinated European framework for identifying and evaluating repurposing candidates for high-priority pathogens. Such a framework could involve close cooperation between HERA, the European Medicines Agency, research institutions, and pharmaceutical manufacturers. Through early mapping of therapeutic classes and potential repurposing opportunities, the EU could significantly shorten the time required to deploy therapeutic interventions during future health emergencies.

In addition, the growing availability of real-world clinical data and patient registries may support faster evaluation of repurposed medicines in emergency contexts. When combined with adaptive clinical trial designs and coordinated regulatory guidance, these data sources could contribute to more agile evidence generation during rapidly evolving public health crises.

The role of digital health infrastructure and the enhancement of data analysis models in support of drug repurposing should be examined. The increasing availability of interoperable health data in the European Union, including digital health records, pharmacological databases, and sources of data from real clinical practice (real-world evidence), provides the possibility for a more systematic identification of potential therapeutic applications of medicinal products already approved for use. With the support of AI and machine learning, science can develop new therapeutic signals, thus speeding up the process of hypothesis generation for clinical assessment. In this context, coordinated initiatives at the European level, with the participation of the European Medicines Agency and other key stakeholders, can improve the exchange, standardisation, and analysis of data in a broader context. This type of approach would enhance the scientific basis of drug repurposing strategies, as well as improve the development of a more proactive and data-based model for health crisis preparedness in the European Union.

Although HERA is not a regulatory authority, its activities already intersect with key components of drug repurposing within the EU health security framework. In particular, HERA contributes to the identification and prioritisation of medical countermeasures through threat assessment and horizon scanning, which forms the basis for selecting candidate therapeutics for further development.

This role is complemented by the implementation of the EU Medical Countermeasures Strategy, which foresees the use of advanced data analytics and artificial intelligence tools to support the identification of promising therapeutic candidates, including existing medicinal products with repurposing potential. Furthermore, HERA supports research and innovation actions through EU-level funding instruments, thereby indirectly facilitating the generation of clinical evidence required for repurposing [38].

Although HERA is not a regulatory authority, its activities already include substantial and operational financial support for the development of medical countermeasures across the full innovation lifecycle. Funding is channelled through multiple established instruments, including EU4Health, Horizon Europe, rescEU, and HERA Invest, which collectively support research, clinical trials, manufacturing capacity, and deployment of therapeutics and vaccines. In practice, HERA has already financed clinical research on therapeutics, supported the development of broad-spectrum antivirals, and launched targeted calls for proposals aimed at strengthening preparedness against emerging health threats. Moreover, the HERA Invest instrument provides dedicated financing for companies in early and late clinical development phases, addressing structural market failures that are particularly relevant for repurposed medicinal products lacking commercial incentives. These concrete funding activities demonstrate that HERA plays an active, upstream role in enabling the generation of clinical evidence and supporting the therapeutic development pipeline, including pathways that are directly applicable to drug repurposing [38,40].

### 4.5. Study Limitations

Several limitations of this study should be acknowledged. First, the analysis is based on a narrative literature review and policy analysis, rather than a systematic review. As a result, although the study draws upon a broad range of scientific publications and policy documents, it may not capture all relevant literature related to drug repurposing and EU health crisis governance.

Second, the institutional architecture of the European Union’s health security framework is still evolving. Many of the mechanisms associated with the Health Emergency Preparedness and Response Authority (HERA), including industrial preparedness initiatives and crisis coordination instruments, remain in relatively early stages of implementation. Consequently, empirical evidence regarding their long-term effectiveness is still limited.

Third, this study focuses primarily on institutional and policy dimensions of drug repurposing within the EU health crisis preparedness framework. It does not provide a quantitative assessment of specific therapeutic outcomes or clinical effectiveness of repurposed medicines. Future research could complement this policy-oriented analysis by examining clinical trial data, real-world evidence, and regulatory outcomes associated with repurposing strategies during health emergencies.

## 5. Conclusions

The experience of recent global health crises has demonstrated the critical importance of rapid access to effective medical countermeasures. Within this context, drug repurposing represents a promising strategy for accelerating the availability of therapeutic options during the early stages of emerging health threats.

Compared with the development of novel medicinal products, repurposing offers several potential advantages, including shorter development timelines, lower research costs, and the possibility of leveraging existing manufacturing infrastructure and regulatory knowledge. When combined with coordinated clinical research and appropriate regulatory oversight, repurposing strategies may therefore provide valuable support for emergency response efforts.

At the policy level, the evolving institutional framework of the European Health Union creates new opportunities for integrating drug repurposing into EU health crisis preparedness strategies. In particular, the Health Emergency Preparedness and Response Authority (HERA) may serve as a key platform for coordinating threat intelligence, regulatory expertise, industrial preparedness, and procurement mechanisms across Member States.

The findings of this study suggest that drug repurposing could evolve from an ad hoc response mechanism into a more institutionalised component of EU health security policy. Achieving this transition would require stronger coordination between European institutions, regulatory agencies, research networks, and pharmaceutical manufacturers. In addition, systematic mapping of repurposing opportunities, the integration of real-world clinical data, and the development of adaptive regulatory pathways could further enhance the EU’s ability to deploy therapeutic interventions rapidly during future health emergencies.

Strengthening the role of drug repurposing within the EU preparedness framework could therefore contribute to improving pharmaceutical resilience, reducing strategic dependencies, and enhancing the overall capacity of the European Union to respond effectively to future health crises.

## Data Availability

No new data were created or analysed in this study. Data sharing is not applicable to this article.

## Abbreviations

The following abbreviations are used in this manuscript:

HERA	Health Emergency Preparedness and Response Authority
MCM	Medical Countermeasures
MS	Member States
CBRN	Chemical, Biological, Radiation and Nuclear
RWE	Real World Evidence
EMA	European Medicines Agency
